# Genetic Alterations in Glioma

**DOI:** 10.3390/cancers3011129

**Published:** 2011-03-07

**Authors:** Linda B. C. Bralten, Pim J. French

**Affiliations:** Department of Neurology, Erasmus University Medical Center, Erasmus University Rotterdam, Dr Molewaterplein 50, 3000 CA, Rotterdam, the Netherlands; E-Mail: l.bralten@erasmusmc.nl

**Keywords:** glioma, EGFR, PTEN, CDKN2A, RB, TP53, LOH 1p19q, IDH1, pathway

## Abstract

Gliomas are the most common type of primary brain tumor and have a dismal prognosis. Understanding the genetic alterations that drive glioma formation and progression may help improve patient prognosis by identification of novel treatment targets. Recently, two major studies have performed in-depth mutation analysis of glioblastomas (the most common and aggressive subtype of glioma). This systematic approach revealed three major pathways that are affected in glioblastomas: The receptor tyrosine kinase signaling pathway, the TP53 pathway and the pRB pathway. Apart from frequent mutations in the *IDH1/2* gene, much less is known about the causal genetic changes of grade II and III (anaplastic) gliomas. Exceptions include *TP53* mutations and fusion genes involving the *BRAF* gene in astrocytic and pilocytic glioma subtypes, respectively. In this review, we provide an update on all common events involved in the initiation and/or progression across the different subtypes of glioma and provide future directions for research into the genetic changes.

## 1. Introduction

Gliomas are the most common primary brain tumor in adults (incidence 5.97 per 100,000, CBTRUS statistical report 2010, http://www.cbtrus.org/) and can be subdivided based on their histological appearance into an astrocytic (A), oligodendroglial (OD), or oligoastrocytic (OA) lineage. They can be further subclassified into grades: I (pilocytic astrocytomas, PA), II (low grade), III (anaplastic) and IV (glioblastoma, GBM), depending on the number of malignant features present (high cellularity, nuclear atypia, mitosis, necrosis, and endothelial proliferation). Secondary GBMs are those that have progressed from lower grade gliomas, whereas primary GBMs arise *de novo*.

The prognosis of glioma patients varies between the different histological subtypes and grades; for example, patients with grade II OD have the longest survival (median survival 11.5 years) whereas patients with a glioblastoma (the most common subtype of glioma) have a median survival of only 4.9 months [1]. Pilocytic astrocytomas are the only subtype of glioma with a favorable prognosis (94–96% five year survival) (CBTRUS statistical report 2010; [1]).

Current treatment options involve a combination of surgical resection, radiotherapy and chemotherapy. Complete surgical resection of gliomas is virtually impossible due to their invasive growth pattern. Radiotherapy is effective. However, the infiltrating cells can only be reached by whole brain irradiation where the benefits of radiotherapy may not outweigh the side effects caused by radiation damage. Temozolomide has thusfar proven effective in improving the median overall survival in glioblastoma by three months [2], and is most effective in those in which the *MGMT* promoter is hypermethylated [3]. Adjuvant procarbazine, CCNU (lomustine) and vincristine (PCV) improved progression free survival but not overall survival in anaplastic oligodendrogliomas and oligoastrocytomas [4,5]. In these studies, tumors harboring loss of 1p and 19q chromosomal arms comprise a favorable subgroup. In spite of these advances, the prognosis of gliomas remains poor and therefore other strategies have to be developed. A better understanding of the genes (and their associated molecular pathways) involved in gliomagenesis and/or progression may reveal new options for targeted therapy.

## 2. Glioblastoma

**Scheme 1.**
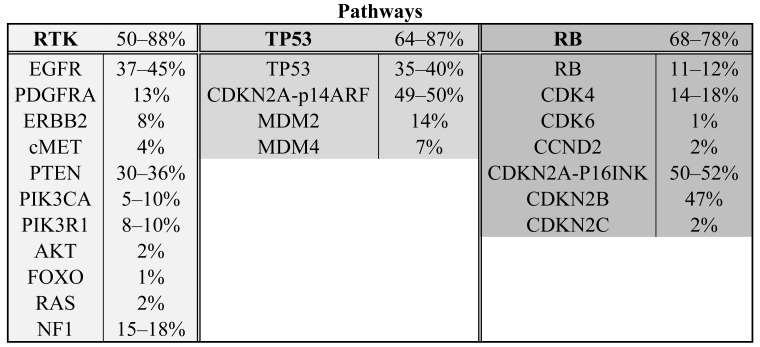
The three major pathways affected in a high percentage of glioblastomas and the most common genes affected in those pathways. Following the gene names, the percentages of genetic alterations found in glioblastoma are depicted.

The Cancer Genome Atlas project has published mRNA expression data and DNA copy number alteration data of 206 GBMs and has sequenced >600 genes in 91 GBMs [6]. The project is still ongoing and ultimately aims to include data from 500 gliomas, but already has shown the importance of a systematical approach and high sample numbers. Combining all detected homozygous deletions, focal amplifications and validated somatic nucleotide substitutions, they found three major pathways affected in a high percentage of glioblastomas: receptor tyrosine kinase signaling (altered in 88% of the GBMs), TP53 signaling (altered in 87%) and the pRB tumor suppressor pathway (altered in 78%). Novel genes in those pathways include the NF1 tumor suppressor gene and PIK3R1. Another large study on 22 GBMs sequenced all protein coding genes and performed copy number analysis and expression data analysis on these tumors [7]. All genes affected in more than two tumors were validated in a set of 80 GBMs. Besides confirmation of affected oncogenes and tumor suppressor genes in the formerly mentioned three pathways (50%, 64% and 68%, respectively) (see Scheme 1 and Table 1), they found the *IDH1* gene to be mutated in 12% of the GBMs [7]. These mutations occurred in younger patients with mostly secondary GBMs and such tumors had a relatively favorable prognosis [7,8]. (For a more elaborate discussion on *IDH1* see below).

### 2.1. Copy Number Alterations

Copy number amplifications are more frequent in GBMs than in lower grade gliomas [9,10]. A distinction can be made between focal, high copy number amplifications (e.g., ≥7n) and larger, intermediate copy number amplifications (e.g., 3n). High copy amplicons often occur in regions with known oncogenes (*EGFR*, *MDM2* and *CDK4*), but may also occur in other regions [11] (for a summary see [12]). Frequent intermediate copy number gains or losses include trisomy of chromosome 7 (42%) and monosomy of chromosome 10 (58%) [6,13,14]. The genes involved in gliomagenesis and/or progression on these chromosomes (except for *PTEN*) remain to be determined. GBMs with trisomy of chromosome 7and loss of chromosome 10 have a poor prognosis [6,13,14]. In general, aCGH data can identify three genetic GBM subgroups; one with gain of chromosome 7 and loss of chromosome 10, one with only chromosome10 loss and one without gain of chromosome 7 or loss of chromosome 10 [15].

### 2.2. Receptor Tyrosine Kinase Signaling

The receptor tyrosine kinase (RTK) signaling pathway is involved in the translation of growth factor signals into increased proliferation and survival. The most frequently altered gene in the RTK pathway is *EGFR*. It is amplified in up to 45% of glioblastomas and results in an increase in mRNA and protein expression [6,16,17]. In addition to amplification, *EGFR* is often constitutively activated by variants including *EGFRVIII* in which exons 2–7 are deleted [18]. 3′ truncations are also frequently observed [18]. Both variants are due to intragenic deletions, are thought to occur following *EGFR* amplification and result in constitutively active proteins [19-22]. Although a number of point mutations are also observed in the *EGFR* gene [6,7], the activating mutations in the ATP binding domain observed in lung cancer (NSCLC) are not observed in gliomas [23]. Other RTKs may also be affected in GBM and include *PDGFRA* (amplified in 13%), *ERBB2* (mutated in 8%) and *cMET* (amplified in 4%) [6].

RTKs signal (a.o.) through phosphoinositide 3 kinases (PI3 kinases) that phosphorylate phosphatidylinositol (4,5)-bisphosphate (PIP2) to phosphatidylinositol (3,4,5)-trisphosphate (PIP3). This reaction is reversed by PTEN. Of the PI3 kinases, *PIK3CA* and its adaptor protein *PIK3R1* are most frequently mutated in GBM (15–27%) [6,7,24,25]. *PTEN* is homozygously deleted in 36% of GBMs and infrequent mutations in downstream PIP3 targets have been identified in *AKT* and *FOXO* (2% AKT amplification, 1% inactivating FOXO mutation) [6].

Ras is another important protein activated by receptor tyrosine kinases and a key regulator of tumorigenesis. *Ras* mutations (*N-Ras*, *H-Ras*, *KRas*) occur infrequently in GBM (2% activating *Ras* mutations) [6]. However, the *NF1* gene, which encodes for the Ras inhibiting protein neurofibromin 1 (a RasGAP), is frequently inactivated in GBM (15-18% inactivating mutations or homozygous deletions) [6,7].

### 2.3. TP53 Signaling

TP53 signaling is important in apoptosis, cellular senescence and cell cycle arrest in response to DNA damage. Most tumor types need to circumvent or shut down the TP53 pathway. *TP53* heterozygous dominant negative point mutations and homozygous deletions are common in GBM (35–40%) [6,7]. Two TP53 inhibitors, *MDM2* and *MDM4*, that are involved in the ubiquitinylation and degradation of TP53, are amplified in 14% and 7% of the glioblastomas, respectively [6]. The *CDKN2A* locus is also part of the TP53 pathway and is frequently deleted or inactivated in glioblastomas (49–50%) [6,7]. One of the two genes that can be expressed from the *CDKN2A* locus is *P14ARF*, which is an inhibitor of MDM2. The *CDKN2A* locus also encodes for p16INK4A which is part of the pRB signaling pathway (see below)

### 2.4. RB Signaling

The retinoblastoma protein (pRB) is a major protein involved in cell cycle progression from G1 to S phase. In the hypophosphorylated state pRB binds to the transcription factor E2F, thereby preventing cell cycle progression. Phosphorylated pRB does not associate with E2F, which results in cell cycle progression. The *pRB* gene is homozygously deleted or mutated in 11–12% of the GBMs [6,7]. Interestingly, *CDK4*, *CDK6* and *CCND2* phosphorylate pRB and are amplified in 14–18%, 1% and 2% of glioblastomas, respectively [6,7]. Conversely, CDKN2A/p16INK4A, *CDKN2B* and *CDKN2C*, inhibit the different CDKs and are frequently inactivated in GBM (homozygously deleted or mutated in 50–52%, homozygously deleted in 47%, homozygously deleted in 2%, respectively) [6,7].

## 3. Grade II and III Glioma

In general, only few frequent genetic changes have been identified in lower grade gliomas (Table 1). Larger chromosomal aberrations include combined loss of 1p19q in grade II and III oligodendroglioma (40–69%) and oligoastrocytoma (44–48%). Such losses are far less common in astrocytoma (0–11%) [1,26,27]. The remarkably high frequency of LOH of 1p and 19q suggests the remaining arms harbor yet to be identified tumor suppressor genes (Knudson two-hit hypothesis [28]). Frequent mutations have been identified in *IDH1* (all grade II and III gliomas, see below) and *TP53* genes (predominantly astrocytic). *TP53* mutations and LOH of 1p 19q are mutually exclusive [1], thereby distinguishing two different pathways of glioma development. Complete hemizygous losses of 1p are tightly associated with 19q loss and oligodendroglial phenotype and predict longer overall and progression free survival [29]. However, partial 1p deletions are mainly observed in astrocytic tumors, are not associated with 19q loss and have a negative prognostic value [29].

Based on these genetic changes, three large groups can be genetically characterized in low grade and anaplastic gliomas: tumors with *TP53* mutations and *IDH1/2* mutations (32%), tumors with LOH 1p19q and *IDH1/2* mutations (37%) and tumors with only *IDH1/2* mutations (17%) [30]. These molecular changes segregate with the distinct histological subgroups of glioma: for example, most of the diffuse astrocytoma have *TP53* and *IDH1/2* mutation and most of the oligodendrogliomas have LOH 1p19q and *IDH1/2* mutation. The oligoastrocytomas were more equally distributed among the three different groups (33%, 34% and 19% respectively) [30].

### IDH1/IDH2

*IDH1* mutations were initially discovered in GBMs by Parsons *et al.* [7]. However, *IDH1* mutations were detected at much higher frequencies (over 70%) in grade II and II gliomas [31-35].; Mutations in the homologous *IDH2* gene were also identified (around 5%), predominantly in oligodendroglial tumors [32,35]. *IDH1* mutations are an early event in tumorigenesis [34], are an independent favorable prognostic marker in gliomas and are closely associated with1p19q codeletion and *MGMT* methylation status [36]. *IDH1/2* mutations are heterozygous missense mutations affecting highly conserved arginines that are involved in substrate binding. Wildtype IDH converts isocitrate into alpha-ketoglutarate, whereas mutant IDH enzymes have reduced ability to catalyze this reaction [35]. Instead, mutant IDH enzymes have gained the ability to convert alpha-ketoglutarate into D-2-hydroxyglutarate [37]. The oncogenic function and the molecular pathway of IDH1/2 and D-2-hydroxyglutarate are not fully understood yet. However, a recent study suggests that *IDH1/2* mutations result in an increase in global methylation [38] and IDH1/2 mutations are associated with a more hypermethylated DNA methylation profile [39,40]. It is therefore possible that IDH1/2 mutations are involved in oncogenesis by the inactivation of tumor suppressor genes following promoter hypermethylation.

## 4. Pilocytic Astrocytoma

The majority of pilocytic astrocytomas are cytogenetically normal [41] except for a small tandem dulication of 2 Mb at 7q34 (66%) [42]. This duplication results in a fusion gene incorporating the kinase domain of *BRAF* to *KIAA1549* (exon 1-16/15) [42]. This fusion gene produces a constitutively active BRAF, which is able to transform NIH3T3 cells [42]. Activating *BRAF* point mutations (V600E/ins 3 bp at 598) can also occur in pilocytic astrocytoma [42,43].

## 5. Genetic Alterations in Molecular Subtypes

Current classification of gliomas is largely based on histological appearance. However, histological classification of gliomas is troublesome and subject to interobserver-variation [44-47]. Gliomas may also be classified based on their similarities in gene expression profiles [48-50]. Such classification correlates better with survival than histological classification of gliomas [48]. Distinct genetic changes also segregate into the different molecular subtypes of glioma [48,50]. For example, EGFR amplifications are predominantly found in classical and neural subtypes [50] or in cluster 18 gliomas [48]. *IDH1* mutations segregate in proneural type GBMs [50] or in gliomas containing predominantly secondary GBMs (Cluster 22) as well as in gliomas with more favorable prognosis (Clusters 9 and 17) [48].

## 6. Epigenetic Changes

Methylation in cancer often occurs in the promoter regions of tumor suppressor genes [51,52]. Inactivation of gene expression by promoter methylation thus contributes to tumor formation as the second “hit” in tumor suppressor genes (Knudson's two-hit hypothesis [28]). Several groups have therefore performed genome wide methylation profiling in GBMs and have identified a subset of tumors that have more favorable prognosis [40,53]. These tumors show an overall increase in DNA methylation at CpG sites (CIMP; CpG island methylator phenotype) [40]. It remains to be determined whether reversal of CIMP status can be used as a treatment for gliomas.

## 7. Future Perspectives

Large scale sequencing efforts, such as those described in this review, have revealed an unprecedented insight into the biology of gliomas. The rapid development of novel sequencing techniques will lead to even more genetic data in the next decade. It can therefore be expected that virtually all genetic changes in all glioma subtypes will be identified in the near future. However, not all genetic changes in gliomas (or other tumors) are causal for the disease; mutations can arise during cell division and are then found throughout the tumor due to clonal expansion [54]. In fact, the majority of somatic mutations in cancer may be such “passenger” mutations [54]. Distinguishing such “passenger” mutations from the causal “driver” mutations is therefore required.

One way to distinguish driver from passenger mutations is by frequency analysis: causal genetic changes are thought to occur at a higher incidence than predicted by chance. However, few genetic changes are recurrent events (so-called “mountains” in the genomic landscape of [55]); only a handful of genes are mutated at frequencies >10% not only in gliomas [6,7,54] but also in many other cancer types [56,57]. These studies show that many more genes are mutated at low frequencies (so-called “hills”). Such infrequent candidates have been demonstrated to contribute to tumor formation and/or progression (see e.g., [58]). Future research will thus require distinguishing “drivers” from “passengers” on infrequently mutated genes.

Eventually, the knowledge of these genetic alterations can be used for the development of targeted therapy. However, many infrequent “hills” could indicate that each tumor has its own unique spectrum of causal genetic changes. Treatments aimed at targeting these individual genetic changes may therefore be difficult. Nevertheless, it is likely that different genetic changes are part of a select set of molecular pathways. Therefore, pathway inhibition or reactivation can be used to target a broader range of tumors. In the future, it is likely that individual cancer genomes will therefore be sequenced to direct targeted therapies. Such practice does require a further increase in sequencing capacity and speed and dedicated data analysis pipelines.

## Figures and Tables

**Table 1.** Genes or regions with the most characteristic genetic alterations in glioma subtypes. * % are calculated on a mixture of primary and secondary glioblastoma. LOH: loss of heterozygosity; PA: Pilocytic Astrocytoma.

**Ref.**		[7]*	[6]*	[43]	[31]	[32]	[27]	[1]*
**Tumor Type**								
**PA**	BRAF			66%				
**Oligodendroglioma**	IDH1/2				69%	74.7–86.7%		
	LOH 1p19q						40%	69%
**Oligoastrocytoma**	IDH1/2				78%	72.3–82.9%		
	LOH 1p19q						48%	45%
	TP53							44%
**Astrocytoma**	IDH1/2				68%	64.9–73.6%		
	TP53							53-88%
**Sec Glioblastoma**	IDH1/2	12%			88%			
	TP53	40%	35%					31%
**Prim Glioblastoma**	EGFR	37%	45%					34%
	CDKN2A	50%	53%					31%
	PTEN	30%	36%					24%

## References

[1] Ohgaki H., Kleihues P. (2005). Population-based studies on incidence, survival rates, and genetic alterations in astrocytic and oligodendroglial gliomas. J. Neuropathol. Exp. Neurol..

[2] Stupp R., Mason W.P., van den Bent M.J., Weller M., Fisher B., Taphoorn M.J., Belanger K., Brandes A.A., Marosi C., Bogdahn U., Curschmann J., Janzer R.C., Ludwin S.K., Gorlia T., Allgeier A., Lacombe D., Cairncross J.G., Eisenhauer E., Mirimanoff R.O. (2005). Radiotherapy plus concomitant and adjuvant temozolomide for glioblastoma. N. Engl. J. Med..

[3] Hegi M.E., Diserens A.C., Gorlia T., Hamou M.F., de Tribolet N., Weller M., Kros J.M., Hainfellner J.A., Mason W., Mariani L., Bromberg J.E., Hau P., Mirimanoff R.O., Cairncross J.G., Janzer R.C., Stupp R. (2005). Mgmt gene silencing and benefit from temozolomide in glioblastoma. N. Engl. J. Med..

[4] van den Bent M.J., Carpentier A.F., Brandes A.A., Sanson M., Taphoorn M.J., Bernsen H.J., Frenay M., Tijssen C.C., Grisold W., Sipos L., Haaxma-Reiche H., Kros J.M., van Kouwenhoven M.C., Vecht C.J., Allgeier A., Lacombe D., Gorlia T. (2006). Adjuvant procarbazine, lomustine, and vincristine improves progression-free survival but not overall survival in newly diagnosed anaplastic oligodendrogliomas and oligoastrocytomas: A randomized european organisation for research and treatment of cancer phase iii trial. J. Clin. Oncol..

[5] Giannini C., Burger P.C., Berkey B.A., Cairncross J.G., Jenkins R.B., Mehta M., Curran W.J., Aldape K. (2008). Anaplastic oligodendroglial tumors: Refining the correlation among histopathology, 1p 19q deletion and clinical outcome in intergroup radiation therapy oncology group trial 9402. Brain Pathol..

[6] Cancer Genome Atlas Research N. (2008). Comprehensive genomic characterization defines human glioblastoma genes and core pathways. Nature.

[7] Parsons D.W., Jones S., Zhang X., Lin J.C., Leary R.J., Angenendt P., Mankoo P., Carter H., Siu I.M., Gallia G.L., Olivi A., McLendon R., Rasheed B.A., Keir S., Nikolskaya T., Nikolsky Y., Busam D.A., Tekleab H., Diaz L.A., Hartigan J., Smith D.R., Strausberg R.L., Marie S.K., Shinjo S.M., Yan H., Riggins G.J., Bigner D.D., Karchin R., Papadopoulos N., Parmigiani G., Vogelstein B., Velculescu V.E., Kinzler K.W. (2008). An integrated genomic analysis of human glioblastoma multiforme. Science.

[8] Nobusawa S., Watanabe T., Kleihues P., Ohgaki H. (2009). Idh1 mutations as molecular signature and predictive factor of secondary glioblastomas. Clin. Cancer Res..

[9] Arslantas A., Artan S., Oner U., Muslumanoglu M.H., Ozdemir M., Durmaz R., Arslantas D., Vural M., Cosan E., Atasoy M.A. (2007). Genomic alterations in low-grade, anaplastic astrocytomas and glioblastomas. Pathol. Oncol. Res..

[10] Bralten L.B., Kloosterhof N.K., Gravendeel L.A., Sacchetti A., Duijm E.J., Kros J.M., van den Bent M.J., Hoogenraad C.C., Sillevis Smitt P.A., French P.J. (2010). Integrated genomic profiling identifies candidate genes implicated in glioma-genesis and a novel leo1-slc12a1 fusion gene. Genes Chromosomes Cancer.

[11] Beroukhim R., Getz G., Nghiemphu L., Barretina J., Hsueh T., Linhart D., Vivanco I., Lee J.C., Huang J.H., Alexander S., Du J., Kau T., Thomas R.K., Shah K., Soto H., Perner S., Prensner J., Debiasi R.M., Demichelis F., Hatton C., Rubin M.A., Garraway L.A., Nelson S.F., Liau L., Mischel P.S., Cloughesy T.F., Meyerson M., Golub T.A., Lander E.S., Mellinghoff I.K., Sellers W.R. (2007). Assessing the significance of chromosomal aberrations in cancer: Methodology and application to glioma. Proc. Natl. Acad. Sci. USA.

[12] Rao S.K., Edwards J., Joshi A.D., Siu I.M., Riggins G.J. (2010). A survey of glioblastoma genomic amplifications and deletions. J. Neurooncol..

[13] Hodgson J.G., Yeh R.F., Ray A., Wang N.J., Smirnov I., Yu M., Hariono S., Silber J., Feiler H.S., Gray J.W., Spellman P.T., Vandenberg S.R., Berger M.S., James C.D. (2009). Comparative analyses of gene copy number and mrna expression in glioblastoma multiforme tumors and xenografts. Neuro-oncology.

[14] Schlegel J., Scherthan H., Arens N., Stumm G., Kiessling M. (1996). Detection of complex genetic alterations in human glioblastoma multiforme using comparative genomic hybridization. J. Neuropathol. Exp. Neurol..

[15] Misra A., Pellarin M., Nigro J., Smirnov I., Moore D., Lamborn K.R., Pinkel D., Albertson D.G., Feuerstein B.G. (2005). Array comparative genomic hybridization identifies genetic subgroups in grade 4 human astrocytoma. Clin. Cancer Res..

[16] Lopez-Gines C., Gil-Benso R., Ferrer-Luna R., Benito R., Serna E., Gonzalez-Darder J., Quilis V., Monleon D., Celda B., Cerda-Nicolas M. (2010). New pattern of EGFR amplification in glioblastoma and the relationship of gene copy number with gene expression profile. Mod. Pathol..

[17] Sauter G., Maeda T., Waldman F.M., Davis R.L., Feuerstein B.G. (1996). Patterns of epidermal growth factor receptor amplification in malignant gliomas. Am. J. Pathol..

[18] Frederick L., Eley G., Wang X.Y., James C.D. (2000). Analysis of genomic rearrangements associated with egrfviii expression suggests involvement of alu repeat elements. Neuro-oncology.

[19] Ekstrand A.J., Sugawa N., James C.D., Collins V.P. (1992). Amplified and rearranged epidermal growth factor receptor genes in human glioblastomas reveal deletions of sequences encoding portions of the *N*- and/or *C*-terminal tails. Proc. Natl. Acad. Sci. USA.

[20] Wong A.J., Ruppert J.M., Bigner S.H., Grzeschik C.H., Humphrey P.A., Bigner D.S., Vogelstein B. (1992). Structural alterations of the epidermal growth factor receptor gene in human gliomas. Proc. Natl. Acad. Sci. U S A.

[21] Huang H.S., Nagane M., Klingbeil C.K., Lin H., Nishikawa R., Ji X.D., Huang C.M., Gill G.N., Wiley H.S., Cavenee W.K. (1997). The enhanced tumorigenic activity of a mutant epidermal growth factor receptor common in human cancers is mediated by threshold levels of constitutive tyrosine phosphorylation and unattenuated signaling. J. Biol. Chem..

[22] Grandal M.V., Zandi R., Pedersen M.W., Willumsen B.M., van Deurs B., Poulsen H.S. (2007). Egfrviii escapes down-regulation due to impaired internalization and sorting to lysosomes. Carcinogenesis.

[23] Wood L.D., Calhoun E.S., Silliman N., Ptak J., Szabo S., Powell S.M., Riggins G.J., Wang T.L., Yan H., Gazdar A., Kern S.E., Pennacchio L., Kinzler K.W., Vogelstein B., Velculescu V.E. (2006). Somatic mutations of gucy2f, epha3, and ntrk3 in human cancers. Hum. Mutat..

[24] Mizoguchi M., Nutt C.L., Mohapatra G., Louis D.N. (2004). Genetic alterations of phosphoinositide 3-kinase subunit genes in human glioblastomas. Brain Pathol..

[25] Samuels Y., Wang Z., Bardelli A., Silliman N., Ptak J., Szabo S., Yan H., Gazdar A., Powell S.M., Riggins G.J., Willson J.K., Markowitz S., Kinzler K.W., Vogelstein B., Velculescu V.E. (2004). High frequency of mutations of the pik3ca gene in human cancers. Science.

[26] Bello M.J., Leone P.E., Vaquero J., de Campos J.M., Kusak M.E., Sarasa J.L., Pestana A., Rey J.A. (1995). Allelic loss at 1p and 19q frequently occurs in association and may represent early oncogenic events in oligodendroglial tumors. Int. J. Cancer.

[27] Kraus J.A., Koopmann J., Kaskel P., Maintz D., Brandner S., Schramm J., Louis D.N., Wiestler O.D., von Deimling A. (1995). Shared allelic losses on chromosomes 1p and 19q suggest a common origin of oligodendroglioma and oligoastrocytoma. J. Neuropathol. Exp. Neurol..

[28] Knudson A.G. (1971). Mutation and cancer: Statistical study of retinoblastoma. Proc. Natl. Acad. Sci. USA.

[29] Idbaih A., Marie Y., Pierron G., Brennetot C., Hoang-Xuan K., Kujas M., Mokhtari K., Sanson M., Lejeune J., Aurias A., Delattre O., Delattre J.Y. (2005). Two types of chromosome 1p losses with opposite significance in gliomas. Ann. Neurol..

[30] Kim Y.H., Nobusawa S., Mittelbronn M., Paulus W., Brokinkel B., Keyvani K., Sure U., Wrede K., Nakazato Y., Tanaka Y., Vital A., Mariani L., Stawski R., Watanabe T., De Girolami U., Kleihues P., Ohgaki H. (2010). Molecular classification of low-grade diffuse gliomas. Am. J. Pathol..

[31] Balss J., Meyer J., Mueller W., Korshunov A., Hartmann C., von Deimling A. (2008). Analysis of the idh1 codon 132 mutation in brain tumors. Acta Neuropathol..

[32] Hartmann C., Meyer J., Balss J., Capper D., Mueller W., Christians A., Felsberg J., Wolter M., Mawrin C., Wick W., Weller M., Herold-Mende C., Unterberg A., Jeuken J.W., Wesseling P., Reifenberger G., von Deimling A. (2009). Type and frequency of idh1 and idh2 mutations are related to astrocytic and oligodendroglial differentiation and age: A study of 1,010 diffuse gliomas. Acta Neuropathol..

[33] Kloosterhof N.K., Bralten L.B., Dubbink H.J., French P.J., van den Bent M.J. (2010). Isocitrate dehydrogenase-1 mutations: A fundamentally new understanding of diffuse glioma?. Lancet Oncol..

[34] Watanabe T., Nobusawa S., Kleihues P., Ohgaki H. (2009). Idh1 mutations are early events in the development of astrocytomas and oligodendrogliomas. Am. J. Pathol..

[35] Yan H., Parsons D.W., Jin G., McLendon R., Rasheed B.A., Yuan W., Kos I., Batinic-Haberle I., Jones S., Riggins G.J., Friedman H., Friedman A., Reardon D., Herndon J., Kinzler K.W., Velculescu V.E., Vogelstein B., Bigner D.D. (2009). Idh1 and idh2 mutations in gliomas. N. Engl. J. Med..

[36] Sanson M., Marie Y., Paris S., Idbaih A., Laffaire J., Ducray F., El Hallani S., Boisselier B., Mokhtari K., Hoang-Xuan K., Delattre J.Y. (2009). Isocitrate dehydrogenase 1 codon 132 mutation is an important prognostic biomarker in gliomas. J. Clin. Oncol..

[37] Dang L., White D.W., Gross S., Bennett B.D., Bittinger M.A., Driggers E.M., Fantin V.R., Jang H.G., Jin S., Keenan M.C., Marks K.M., Prins R.M., Ward P.S., Yen K.E., Liau L.M., Rabinowitz J.D., Cantley L.C., Thompson C.B., Vander Heiden M.G., Su S.M. (2009). Cancer-associated idh1 mutations produce 2-hydroxyglutarate. Nature.

[38] Figueroa M.E., Abdel-Wahab O., Lu C., Ward P.S., Patel J., Shih A., Li Y., Bhagwat N., Vasanthakumar A., Fernandez H.F., Tallman M.S., Sun Z., Wolniak K., Peeters J.K., Liu W., Choe S.E., Fantin V.R., Paietta E., Lowenberg B., Licht J.D., Godley L.A., Delwel R., Valk P.J., Thompson C.B., Levine R.L., Melnick A. (2010). Leukemic idh1 and idh2 mutations result in a hypermethylation phenotype, disrupt tet2 function, and impair hematopoietic differentiation. Cancer Cell.

[39] Christensen B.C., Smith A.A., Zheng S., Koestler D.C., Houseman E.A., Marsit C.J., Wiemels J.L., Nelson H.H., Karagas M.R., Wrensch M.R., Kelsey K.T., Wiencke J.K. (2010). DNA methylation, isocitrate dehydrogenase mutation, and survival in glioma. J. Natl. Cancer Inst..

[40] Noushmehr H., Weisenberger D.J., Diefes K., Phillips H.S., Pujara K., Berman B.P., Pan F., Pelloski C.E., Sulman E.P., Bhat K.P., Verhaak R.G., Hoadley K.A., Hayes D.N., Perou C.M., Schmidt H.K., Ding L., Wilson R.K., Van Den Berg D., Shen H., Bengtsson H., Neuvial P., Cope L.M., Buckley J., Herman J.G., Baylin S.B., Laird P.W., Aldape K. (2010). Identification of a cpg island methylator phenotype that defines a distinct subgroup of glioma. Cancer Cell.

[41] Sanoudou D., Tingby O., Ferguson-Smith M.A., Collins V.P., Coleman N. (2000). Analysis of pilocytic astrocytoma by comparative genomic hybridization. Br. J. Cancer.

[42] Jones D.T., Kocialkowski S., Liu L., Pearson D.M., Backlund L.M., Ichimura K., Collins V.P. (2008). Tandem duplication producing a novel oncogenic braf fusion gene defines the majority of pilocytic astrocytomas. Cancer Res..

[43] Jones D.T., Kocialkowski S., Liu L., Pearson D.M., Ichimura K., Collins V.P. (2009). Oncogenic raf1 rearrangement and a novel braf mutation as alternatives to kiaa1549:Braf fusion in activating the mapk pathway in pilocytic astrocytoma. Oncogene.

[44] Hildebrand J., Gorlia T., Kros J.M., Afra D., Frenay M., Omuro A., Stupp R., Lacombe D., Allgeier A., van den Bent M.J., investigators E.B.T.G. (2008). Adjuvant dibromodulcitol and bcnu chemotherapy in anaplastic astrocytoma: Results of a randomised european organisation for research and treatment of cancer phase iii study (eortc study 26882). Eur. J. Cancer.

[45] Kros J.M., Gorlia T., Kouwenhoven M.C., Zheng P.P., Collins V.P., Figarella-Branger D., Giangaspero F., Giannini C., Mokhtari K., Mork S.J., Paetau A., Reifenberger G., van den Bent M.J. (2007). Panel review of anaplastic oligodendroglioma from european organization for research and treatment of cancer trial 26951: Assessment of consensus in diagnosis, influence of 1p/19q loss, and correlations with outcome. J. Neuropathol. Exp. Neurol..

[46] Murphy M., Loosemore A., Ferrer I., Wesseling P., Wilkins P.R., Bell B.A. (2002). Neuropathological diagnostic accuracy. Br. J. Neurosurg..

[47] Scott C.B., Nelson J.S., Farnan N.C., Curran W.J., Murray K.J., Fischbach A.J., Gaspar L.E., Nelson D.F. (1995). Central pathology review in clinical trials for patients with malignant glioma. A report of radiation therapy oncology group 83-02. Cancer.

[48] Gravendeel L.A., Kouwenhoven M.C., Gevaert O., de Rooi J.J., Stubbs A.P., Duijm J.E., Daemen A., Bleeker F.E., Bralten L.B., Kloosterhof N.K., De Moor B., Eilers P.H., van der Spek P.J., Kros J.M., Sillevis Smitt P.A., van den Bent M.J., French P.J. (2009). Intrinsic gene expression profiles of gliomas are a better predictor of survival than histology. Cancer Res..

[49] Li A., Walling J., Ahn S., Kotliarov Y., Su Q., Quezado M., Oberholtzer J.C., Park J., Zenklusen J.C., Fine H.A. (2009). Unsupervised analysis of transcriptomic profiles reveals six glioma subtypes. Cancer Res..

[50] Verhaak R.G., Hoadley K.A., Purdom E., Wang V., Qi Y., Wilkerson M.D., Miller C.R., Ding L., Golub T., Mesirov J.P., Alexe G., Lawrence M., O'Kelly M., Tamayo P., Weir B.A., Gabriel S., Winckler W., Gupta S., Jakkula L., Feiler H.S., Hodgson J.G., James C.D., Sarkaria J.N., Brennan C., Kahn A., Spellman P.T., Wilson R.K., Speed T.P., Gray J.W., Meyerson M., Getz G., Perou C.M., Hayes D.N. (2010). Integrated genomic analysis identifies clinically relevant subtypes of glioblastoma characterized by abnormalities in pdgfra, idh1, egfr, and nf1. Cancer Cell.

[51] Esteller M. (2008). Epigenetics in cancer. N. Engl. J. Med..

[52] Taby R., Issa J.P. (2010). Cancer epigenetics. CA Cancer J. Clin..

[53] Martinez R., Martin-Subero J.I., Rohde V., Kirsch M., Alaminos M., Fernandez A.F., Ropero S., Schackert G., Esteller M. (2009). A microarray-based DNA methylation study of glioblastoma multiforme. Epigenetics.

[54] Greenman C., Stephens P., Smith R., Dalgliesh G.L., Hunter C., Bignell G., Davies H., Teague J., Butler A., Stevens C. (2007). Patterns of somatic mutation in human cancer genomes. Nature.

[55] Wood L.D., Parsons D.W., Jones S., Lin J., Sjoblom T., Leary R.J., Shen D., Boca S.M., Barber T., Ptak J. (2007). The genomic landscapes of human breast and colorectal cancers. Science.

[56] Parsons D.W., Li M., Zhang X., Jones S., Leary R.J., Lin J.C., Boca S.M., Carter H., Samayoa J., Bettegowda C. (2011). The genetic landscape of the childhood cancer medulloblastoma. Science.

[57] Sjoblom T., Jones S., Wood L.D., Parsons D.W., Lin J., Barber T.D., Mandelker D., Leary R.J., Ptak J., Silliman N. (2006). The consensus coding sequences of human breast and colorectal cancers. Science.

[58] Bralten L.B., Gravendeel A.M., Kloosterhof N.K., Sacchetti A., Vrijenhoek T., Veltman J.A., van den Bent M.J., Kros J.M., Hoogenraad C.C., Sillevis Smitt P.A., French P.J. (2010). The caspr2 cell adhesion molecule functions as a tumor suppressor gene in glioma. Oncogene.

